# Supplementary material for: Inflammatory and Cardiovascular Biomarkers to Monitor Fabry Disease Progression
Source: Int J Mol Sci. 2024 May 30;25(11):6024. doi: 10.3390/ijms25116024 (PMC11172779; doi:10.3390/ijms25116024)
Supplement: Supplementary file 1 [file ijms-25-06024-s001.zip › ijms-2981388-supplementary.pdf]

International Journal of
Molecular Sciences

MDPI

Supplementary Information

Inflammatory and cardiovascular biomarkers to monitor Fabry disease progression.

Adrián Alonso-Núñez [1], Tania Pérez-Márquez [1], Marta Alves-Villar [1], Carlos Fernández-Pereira [1], Julián Fernández-Martín [1,2], Alberto Rivera-Gallego [2], Cristina Melcón-Crespo [1,3], Beatriz San Millán-Tejado [1], Aurora Ruz-Zafra [4], Remedios Garofano-López [5], Rosario Sánchez-Martínez [6], Elena García-Payá [6], Manuel López-Mendoza [7], Ignacio Martín-Suárez [8], and Saida Ortolano [1,*]

SI Table S1

Supplementary Table S1. Control patients' cohort description. Activity of α-GalA (µmol/hL) was measured in dried blood spots DBS at basal level of the study. N.A.= not applicable.

Patient ID	Age	Sex	Activity in DBS (µmol/hL)
C1	43	F	4.82±0.7
C2	55	F	5.32±1.80
C3	44	M	7.54±1.32
C4	28	M	3.14±0.05
C5	27	M	4.39±1.16
C6	33	F	3.49±0.94
C7	41	F	3.16±1.19
C8	38	F	2.36±1.10
C9	24	M	3.45±1.50
C10	35	M	5.10±0.13
C11	40	F	2.78±0.68
C12	35	F	6.89±0.54
C13	41	M	2.88±0.28
C14	32	F	5.74±1.18
C15	27	M	3.88±0.23
C16	27	F	5.15±0.36

SI Table S2

Supplementary Table S2. Average Z-score of plasma biomarkers related to inflammatory response in each group of patients, assessed by Luminex-multiplex-ELISA.

Average

Cytokine	Controls (z-score)	Naïves (z-score)	ERT (z-score)
IFN- γ	0,3894389	-0,38695211	-0,47503191
IL-10	0,09462993	-0,24672114	-0,09884109
IL-12p40	0,40037861	-0,17407766	-0,20612811
IL-12p70	-0,17656837	-0,43106565	0,06280232
IL-13	0,43452812	-0,18105338	-0,18105338
IL-17A	1,1468805	-0,2304415	-0,560342
IL-1a	0,40766133	-0,19732581	-0,16070324
IL-9	0,41980445	-0,18994141	-0,16991089
IL-1b	-0,15728639	-0,25683562	0,1729932
IL-4	0,39609054	-0,18631393	-0,15794565

IL-5	0,4115966	-0,17149859	-0,17149859
IL-6	0,31923216	-0,24636601	-0,0952292
MCP-1	-0,34064039	-0,02189766	0,19654388
MIP-1a	0,4115966	-0,17149859	-0,17149859
MIP-1b	-0,38868382	0,2599744	0,12927732
TNFa	-0,50159385	-0,31907106	0,38502027
VEGFA	-0,37932126	-0,04898984	0,0408486
D-dimer	-0,17585961	0,22392752	0,02305728

Detailed statistical analysis of biomarkers validation.

Additional statistical analysis was as follow:

We reanalyzed the quantitative data. To see the distribution of the populations we used the Kolmogorov Smirnov test (represented as KS) and checked if it could be adjusted to a normal distribution (p-value >0.05) or not (p < 0.05).

We compared the distributions of each parameter among groups in order to identify significant differences. Since the values in Fabry Disease patients did not reach significance in the KS test, the medians were compared with nonparametric tests such as the Mann–Whitney test (p-value).

When comparing longitudinal data, we used a non-parametric paired test, the Friedman test (p-value) for the evaluation of each group individually in the three times (Ti, 6 months, and 12 months). Subsequently after that, it was performed the Wilcoxon matched-pairs signed-rank test (p-value) for the detailed bivariate analyses.

To assess the differences between groups (Controls, Naïve and ERT) in each of the time periods, we used the non-parametric Kruskal-Wallis test (p-value). And subsequently the Mann–Whitney (p-value) test as post hoc test for the detailed bivariate analyses. The analysis was performed in IBM SPSS Statistics (Version 26) and are summarized in the SI Tables 3, 4 and 5.

SI Table S3

Supplementary Table S3. *Median values and Interquartile Range (IR) of the proteins in controls (C) and Fabry Disease (FD) patients.*

Parameter	C (IR)	FD (IR)	p-value
ADAMTS-13 (ng/mL)	463.37 (356.91 – 619.02) (N = 16)	549.66 (447.83 – 810.99) (N = 36)	0.104[b]
TNF-α (pg/mL)	7.54 (3.52 - 8.71) (N = 14)	8.43 (7.47 – 14.43) (N = 37)	**0.014[b]**
TNF-α (pg/mL) – No F33	7.54 (3.52 - 8.71) (N = 14)	8.43 (7.46 – 14.01) (N = 36)	**0.018[b]**
GDF-15 (ng/mL)	1.16 (0.45 – 2.77) (N = 16)	1.22 (0.44 – 4.92) (N = 37)	0.535[a]
MCP-1 (ng/mL)	43.31 (38.73 – 56.49) (N = 16)	58.86 (46.33 – 75.63) (N = 37)	**0.025[b]**
MIP-1β (pg/mL)	12.27 (9.48 – 12.51) (N = 14)	12.27 (6.22 – 13.83) (N = 37)	0.746[b]
VEGF (pg/mL)	2.99 (1.53 – 6.34) (N = 16)	14.67 (2.67 – 26.62) (N = 38)	**0.007[b]**

The following tests were applied:

[a]Unpaired Student's t-test.

[b]Mann–Whitney test.

C, control group; FD, Fabry Disease Patients; ADAMTS-13, A disintegrin-like and metalloprotease with thrombospondin type 1 motif no. 13; MCP-1, monocyte chemoattractant protein 1; MIP-1β, macrophage inflammatory protein 1 beta; TNF-α, tumor necrosis factor alpha; GDF-15, Growth Differentiation Factor-15; VEGF, Vascular Endothelial Growth Factor.

SI Table S4

Supplementary Table S4. *p-values of the proteins in controls (C) and Fabry Disease (FD) Naïve patients and Fabry Disease Enzyme Replacement Therapy (FD) patients.*

Parameter	C * (**)			FD Naïve * (**)			FD ERT * (**)		
	Ti vs T6m	Ti vs T12m	T6m vs T12m	Ti vs T6m	Ti vs T12m	T6m vs T12m	Ti vs T6m	Ti vs T12m	T6m vs T12m
ADAMTS-13 (ng/mL)	-	-	-	0.368	0.368	0.368	<0.001 (0.093)	**<0.001 (0.001)**	<0.001 (0.350)
TNF-α (pg/mL)	-	-	-	0.368	0.368	0.368	**0.000 (0.018)**	**0.000 (0.000)**	0.000 (0.199)
TNF-α (pg/mL) – No F33	-	-	-	0.368	0.368	0.368	**0.000 (0.008)**	**0.000 (0.000)**	**0.000 (0.016)**
GDF-15 (ng/mL)	-	-	-	0.607	0.607	0.607	0.046 (1,000)	**0.046 (0.042)**	0.046 (0.392)
MCP-1 (ng/mL)	-	-	-	1.000	1.000	1.000	**0.001(0.014)**	**0.001 (0.002)**	0.001 (1.000)
MIP-1β (pg/mL)	-	-	-	0,317	-	-	0,470	-	-
VEGFA (pg/mL)	-	-	-	0.223	0.223	0.233	**0.004 (1,000)**	**0.004 (0.009)**	**0.004 (0.017)**

The following tests were applied:

*Friedman test

**Wilcoxon matched-pairs signed-rank test

C, control group; FD, Fabry Disease Patients; ADAMTS-13, A disintegrin-like and metalloprotease with thrombospondin type 1 motif no. 13; MCP-1, monocyte chemoattractant protein 1; MIP-1β, macrophage inflammatory protein 1 beta; TNF-α, tumor necrosis factor alpha; GDF-15, Growth Differentiation Factor-15; VEGFA, Vascular Endothelial Growth Factor A.

SI Table S5

Supplementary Table S5. *p-values of the proteins in the three times (Ti, 6 months, and 12 months).*

Parameter	Ti * (**)			T6m * (**)			T12m * (**)		
	C vs FD	C vs ERT	FD vs ERT	C vs FD	C vs ERT	FD vs ERT	C vs FD	C vs ERT	FD vs ERT
ADAMTS-13 (ng/mL)	0.252 (0.323)	0.252 (0.686)	0.252 (1.000)	0.259 (1.000)	0.259 (0.833)	0.259 (0.399)	0.004 (1.000)	**0.004 (0.005)**	0.004 (0.144)
TNF-α (pg/mL)	0.031 (0.610)	**0.031 (0.026)**	0.031 (0.680)	0.854	0.854	0.854	0.002 (0.833)	**0.002 (0.012)**	**0.002 (0.014)**
TNF-α (pg/mL) – No F33	0.015 (0.187)	**0.015 (0.012)**	0.015 (0.609)	0.957	0.957	0.957	0.001 (0.630)	**0.001 (0.008)**	**0.001 (0.011)**

GDF-15 (ng/mL)	0.703	0.703	0.703	0.640	0.640	0.640	0.084	0.084	0.084
MCP-1 (ng/mL)	0.025 (0.831)	**0.025 (0.021)**	0.025 (0.472)	0.259	0.259	0.259	0.074	0.074	0.074
MIP-1β (pg/mL)	0,823	0,823	0,823	0,325	0,325	0,325	-	-	-
VEGF (pg/mL)	0.009 (0.211)	**0.009 (0.006)**	0.009 (0.852)	0.039 (0.097)	0.039 (0.121)	0.039 (1.000)	0.813	0.813	0.813

The following tests were applied:

* Kruskal-Wallis Test

** Mann Whitney

C, control group; FD, Fabry Disease; ERT, Enzyme Replacement Therapy; ADAMTS-13, A disintegrin-like and metalloprotease with thrombospondin type 1 motif no. 13; MCP-1, monocyte chemoattractant protein 1; MIP-1β, macrophage inflammatory protein 1 beta; TNF-α, tumor necrosis factor alpha; GDF-15, Growth Differentiation Factor-15; VEGF, Vascular Endothelial Growth Factor.

SI Table S6

Supplementary Table S6. Average Z-score of plasma biomarkers related to cardiovascular risk factors in each group, assessed by Luminex-multiplex-ELISA.

AVERAGE

Cytokine	Controls (z-score)	Naïves (z-score)	ERT (z-score)
ADAMTS13	0,0522926	1,21025471	-0,43246968
GDF-15	-0,43508193	0,36116183	0,12132491
Myoglobin	-0,21155978	-0,59947211	0,31735725
sICAM-1	-0,12374554	0,79013744	-0,19463163
MPO	-0,70528904	0,42689991	0,24952727
P-selectin	-0,53913356	0,46000806	0,14618263
Lipocalin-2 NGAL	-0,67456502	0,07223974	0,35067843
sVCAM-1	0,22509574	-0,56301393	0,06261812
SAA	-0,26009005	0,42348976	0,00333122

SI Fig. S1

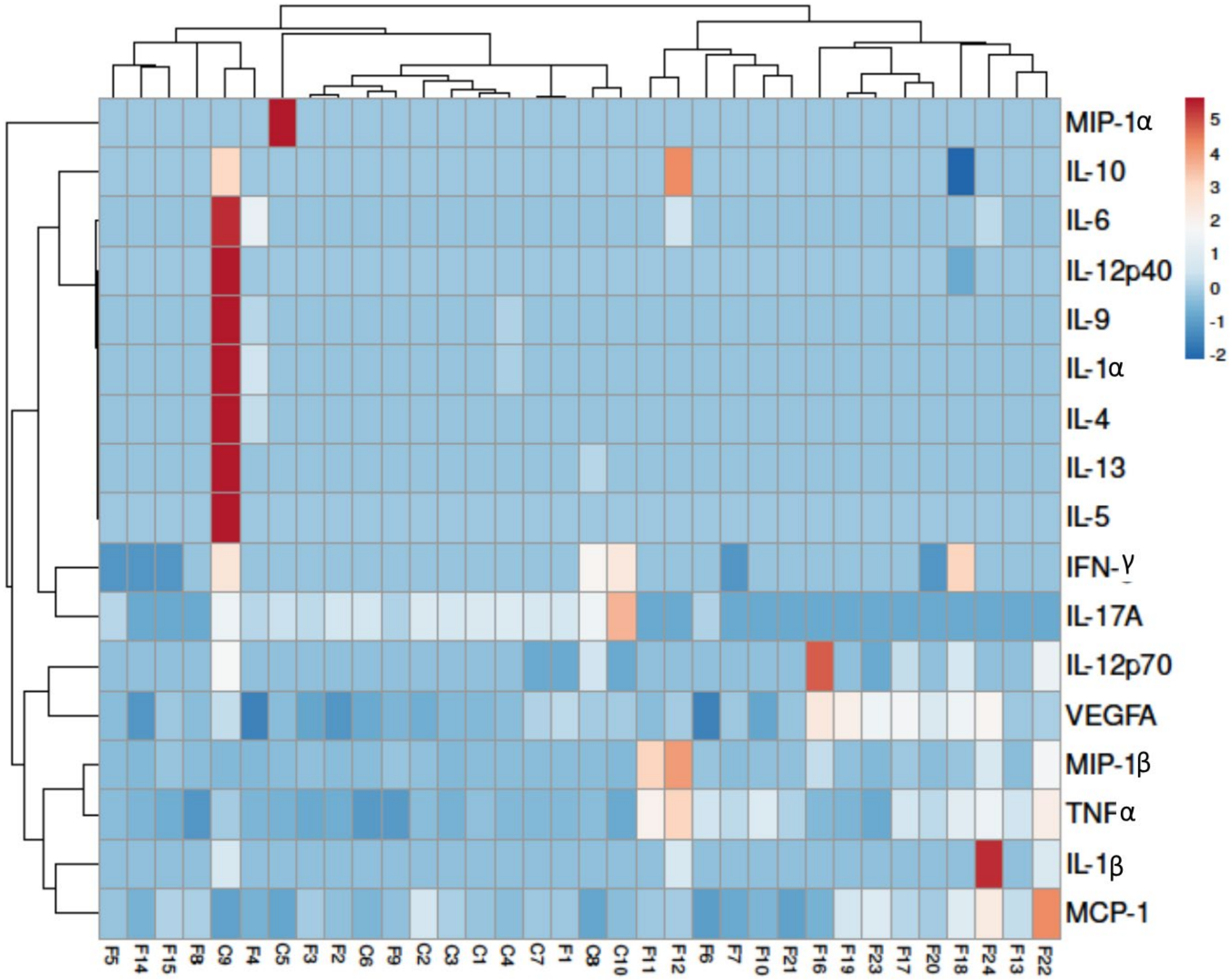

Supplementary Figure S1. Multiplex analysis of Inflammation markers. *Heat map of assessed biomarkers related to inflammatory levels representing Z-score of cytokine concentrations in each sub-cohort, using a color grade scale from dark blue (minimum) to dark red (maximum). Clustering groups for each biomarker and group of patients are indicated by black bars.*

SI Fig. S2

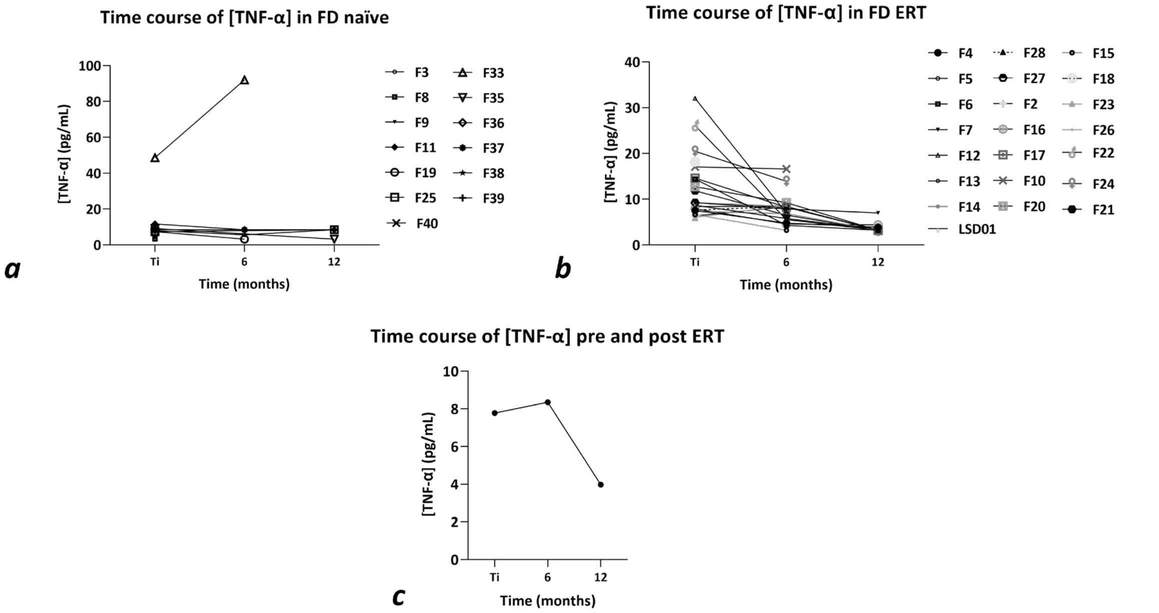

Supplementary Figure S2. Time-course of cardiovascular plasmatic concentration of TNF-α. *It is shown for each patient of the naïve group (a), the ERT treated group (b) and in a patient F1, who started the ERT treatment at Ti (c).*

SI Fig. S3

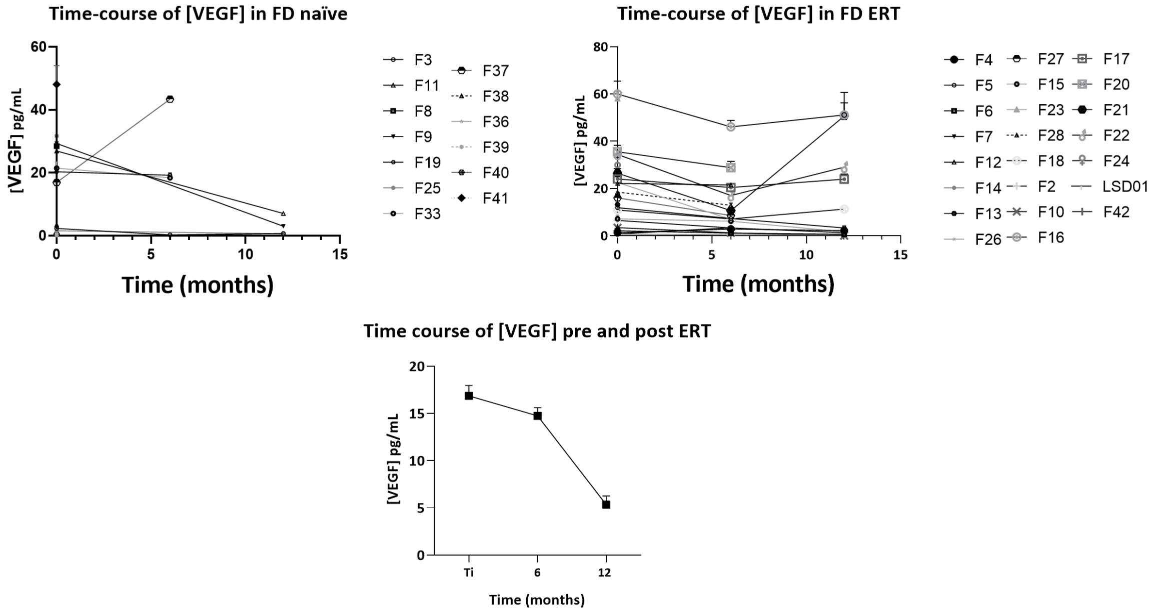

Supplementary Figure S3. Time-course of cardiovascular plasmatic concentration of VEGFA. *It is shown for each patient of the naïve group (a), the ERT treated group (b) and in a patient F1, who started the ERT treatment at Ti (c).*

SI Fig. S4

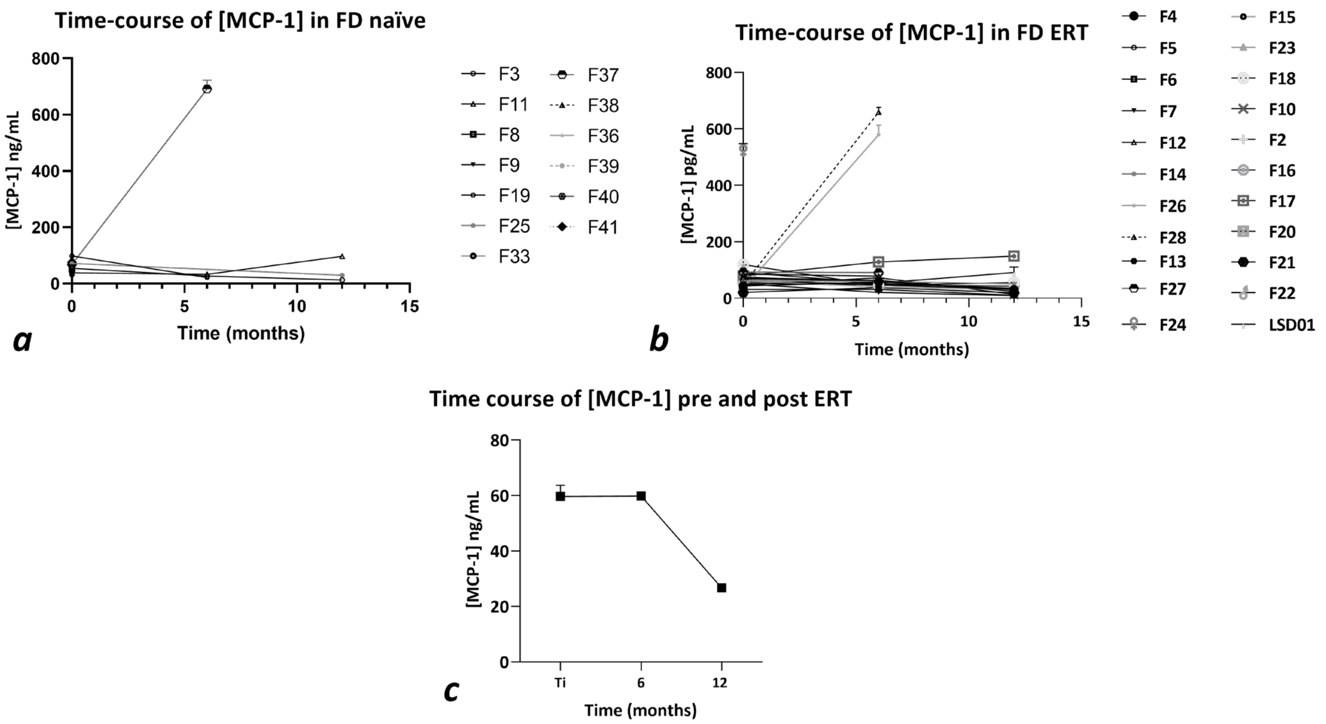

Supplementary Figure S4. Time-course of cardiovascular plasmatic concentration of MCP-1. *It is shown for each patient of the naïve group (a), the ERT treated group (b) and in a patient F1, who started the ERT treatment at Ti (c).*

SI Fig. S5

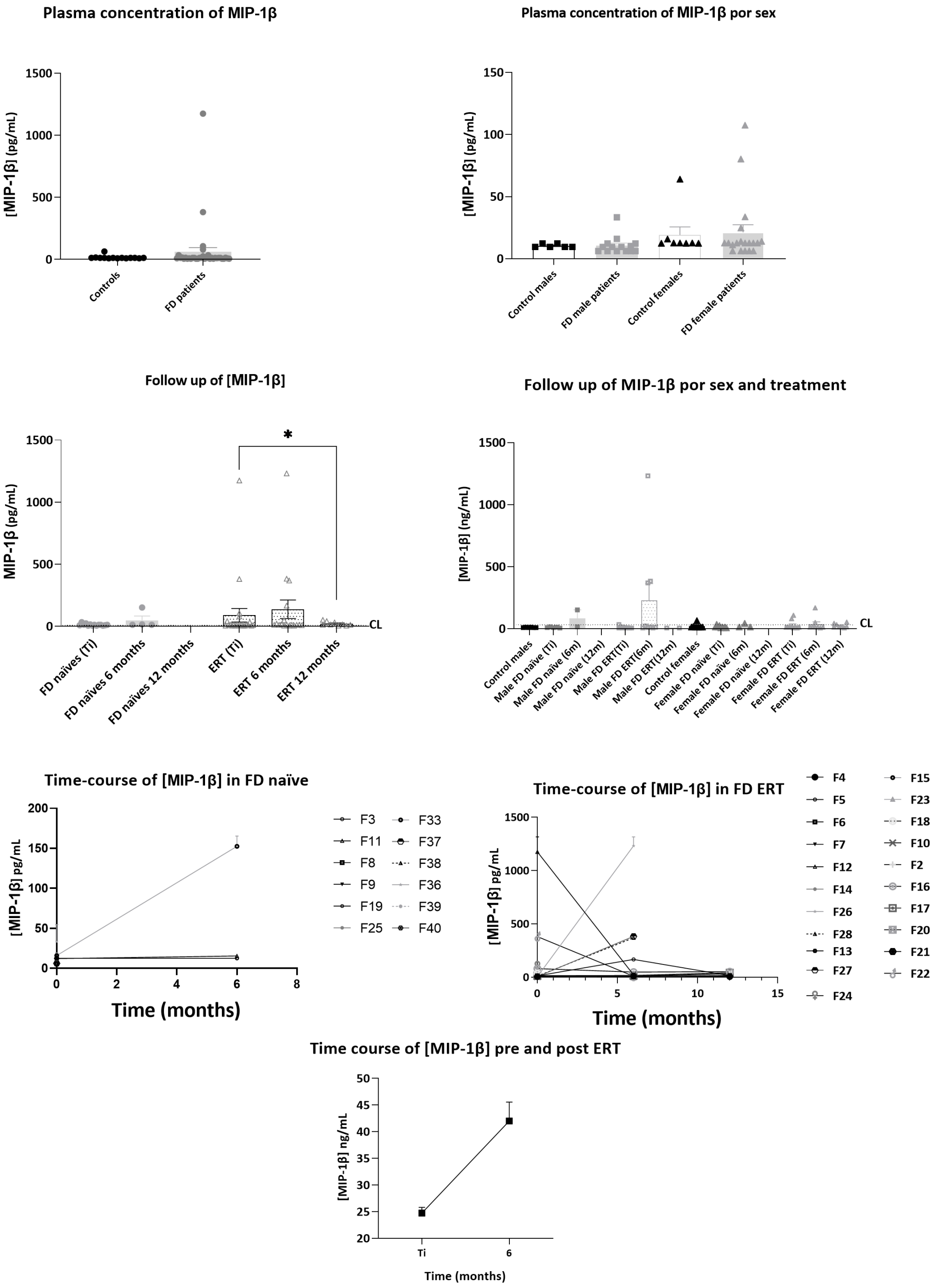

Supplementary Figure S5. Representation of plasmatic concentration of MIP-β in the analyzed cohorts. *(A) Left graph shows histograms representing mean concentration (Mean±SEM) of MIP-β (pg/mL) in FD patients (treated or not with ERT) versus healthy controls. The right diagram represents concentrations of the biomarkers in patients and controls divided por sex. (B) Histograms in the left panel represents the mean concentration (Mean±SEM) of MIP-β in FD naïves and treated patients at different time points (Study onset (Ti), 6 and 12 months after Ti). Dotted line CL represent the mean plasmatic concentration in control subjects. In the right panel, evolution of mean concentration of the biomarker in the treatment groups is discriminated by sex. (C) Time-course of cardiovascular plasmatic concentration of MIP-β is shown for each patient of the naïve group (a), the ERT*

*treated group (b) and in a patient F1, who started the ERT treatment at Ti (c). Statistical significance was assessed with One-way ANOVA non-parametric test (Kruskal-Wallis multiple comparisons, *$p<0.05$).*

SI Fig. S6

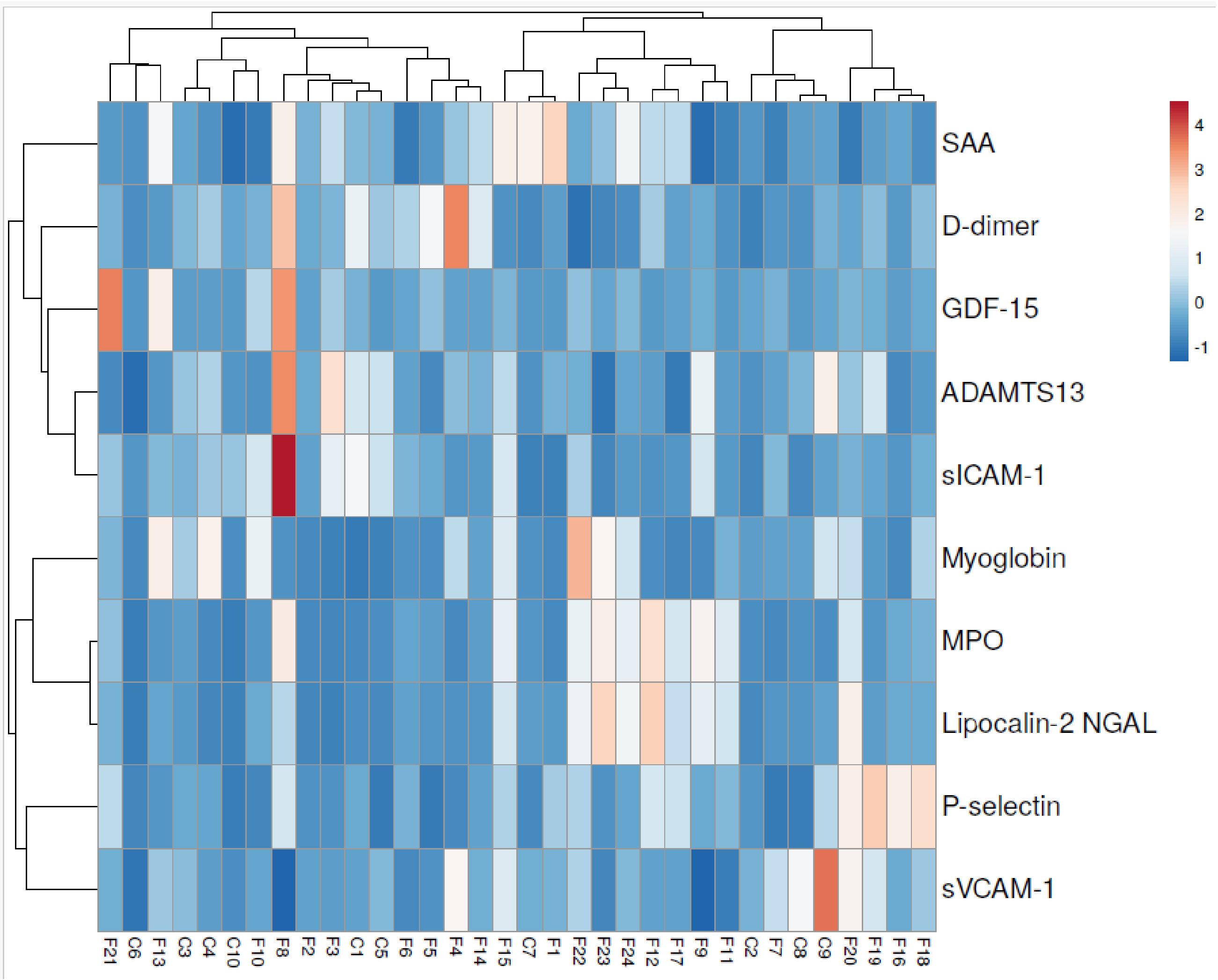

Supplementary Figure S6. Multiplex analysis of cardiovascular risk factors. *Heat map of assessed biomarkers related to inflammatory levels representing Z-score of cytokine concentrations in each sub-cohort, using a color grade scale from dark blue (minimum) to dark red (maximum). Clustering groups for each biomarker and group of patients are indicated by black bars.*

SI Fig. S7

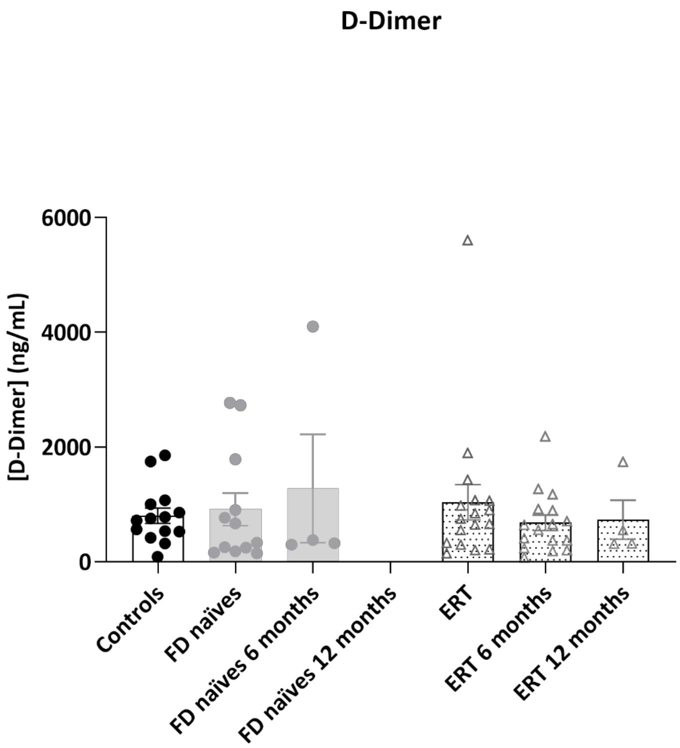

Supplementary Figure S7. Representation of plasmatic concentration of D-Dimer in FD patients and controls. *Histograms represent plasmatic concentration (Mean±SEM) of the protein. Statistical significance was assessed with One-way ANOVA non-parametric test (Kruskal-Wallis multiple comparisons).*

SI Fig. S8

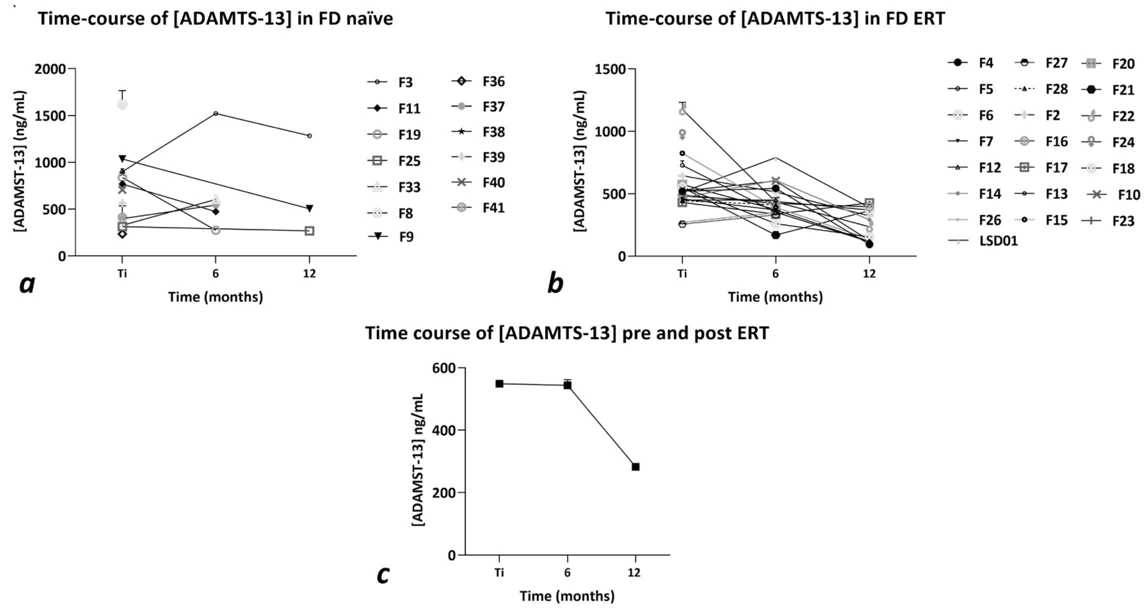

Supplementary Figure S8. Time-course of cardiovascular plasmatic concentration of ADAMTS-13. *It is shown for each patient of the naïve group (a), the ERT treated group (b) and in a patient who started the ERT treatment at Ti (c).*

SI Fig. S9

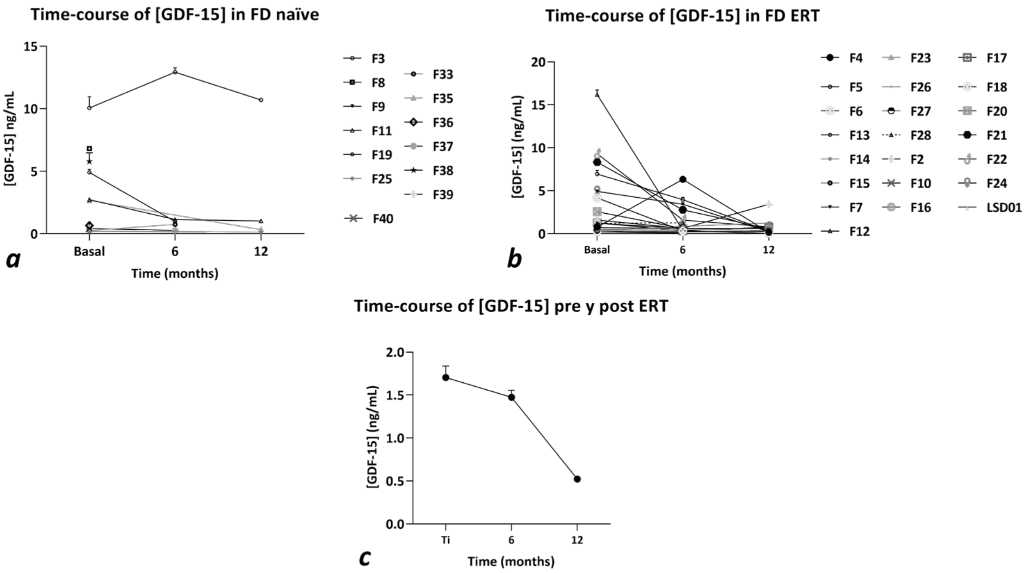

Supplementary Figure S9. Time-course of cardiovascular plasmatic concentration of GDF-15. *It is shown for each patient of the naïve group (a), the ERT treated group (b) and in a patient who started the ERT treatment at Ti (c).*

SI Fig. S10

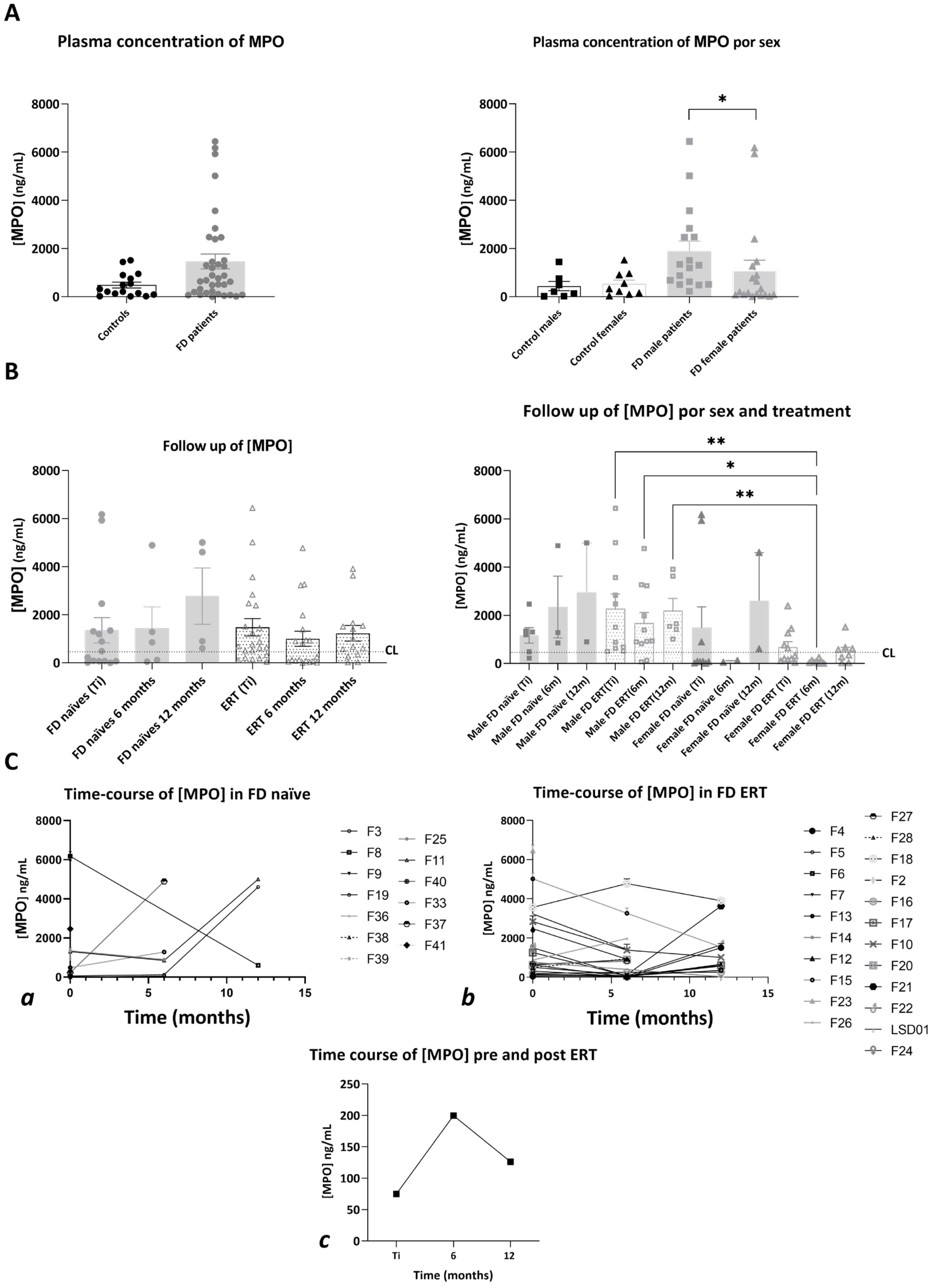

Supplementary Figure S10. Representation of plasmatic concentration of MPO in the analyzed cohorts. *(A) Left graph shows histograms representing mean concentration (Mean±SEM) of MPO (pg/mL) in FD patients (treated or not with ERT) versus healthy controls. The right diagram represents concentrations of the biomarkers in patients and controls divided por sex. (B) Histograms in the left panel represents the mean concentration (Mean±SEM) of MPO in FD naïves and treated patients at*

different time points (Study onset (Ti), 6 and 12 months after Ti). Dotted line CL represent the mean plasmatic concentration in control subjects. In the right panel, evolution of mean concentration of the biomarker in the treatment groups is discriminated by sex. (C) Time-course of cardiovascular plasmatic concentration of MPO is shown for each patient of the naïve group (a), the ERT treated group (b) and in a patient F1, who started the ERT treatment at Ti (c). Statistical significance was assessed with One-way ANOVA non-parametric test (Kruskal-Wallis multiple comparisons).

SI Fig. S11 Correlation of variables analyzed in plasma levels at the beginning of the study

A) ***All patients***

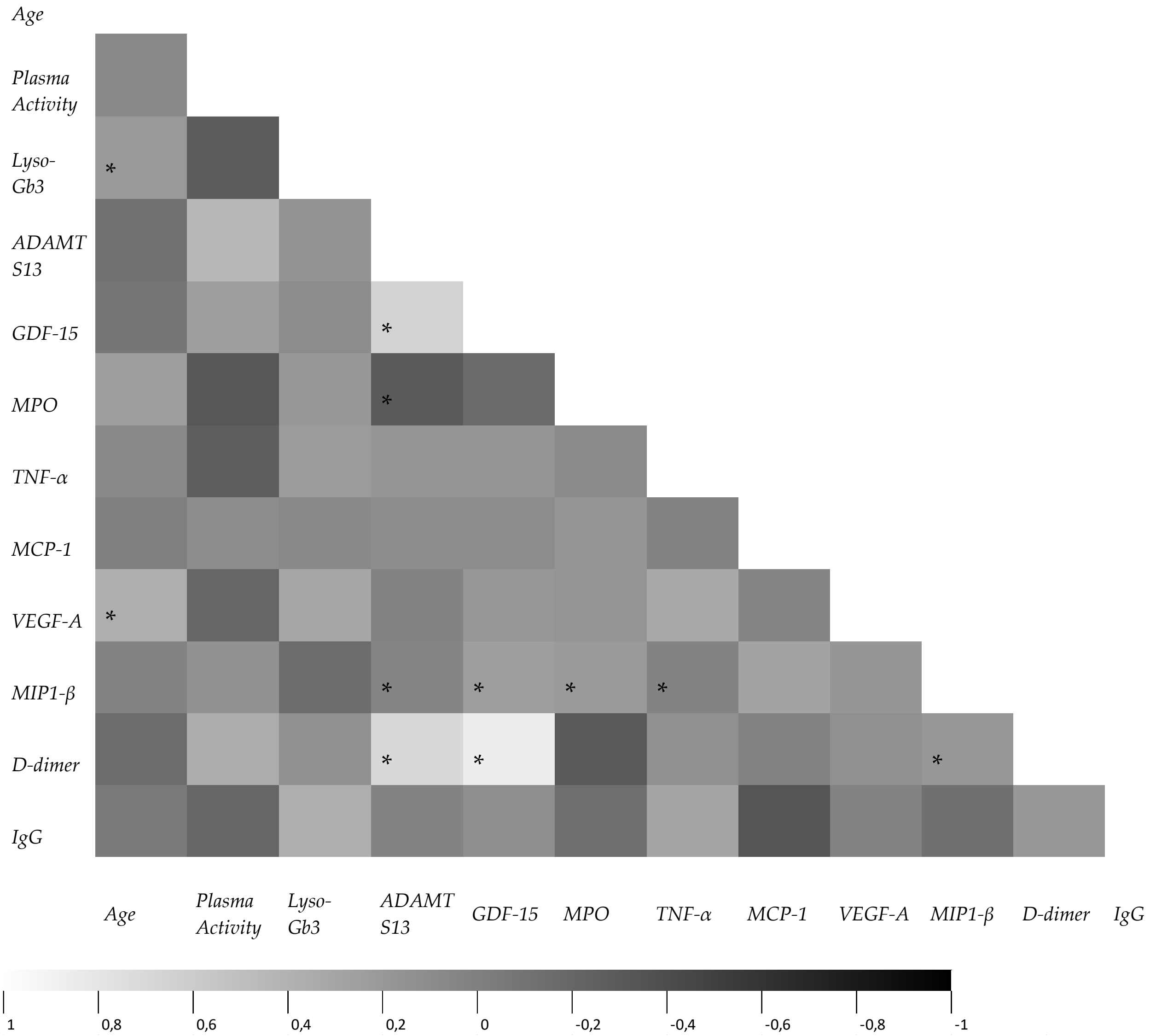

B) ***Male patients***

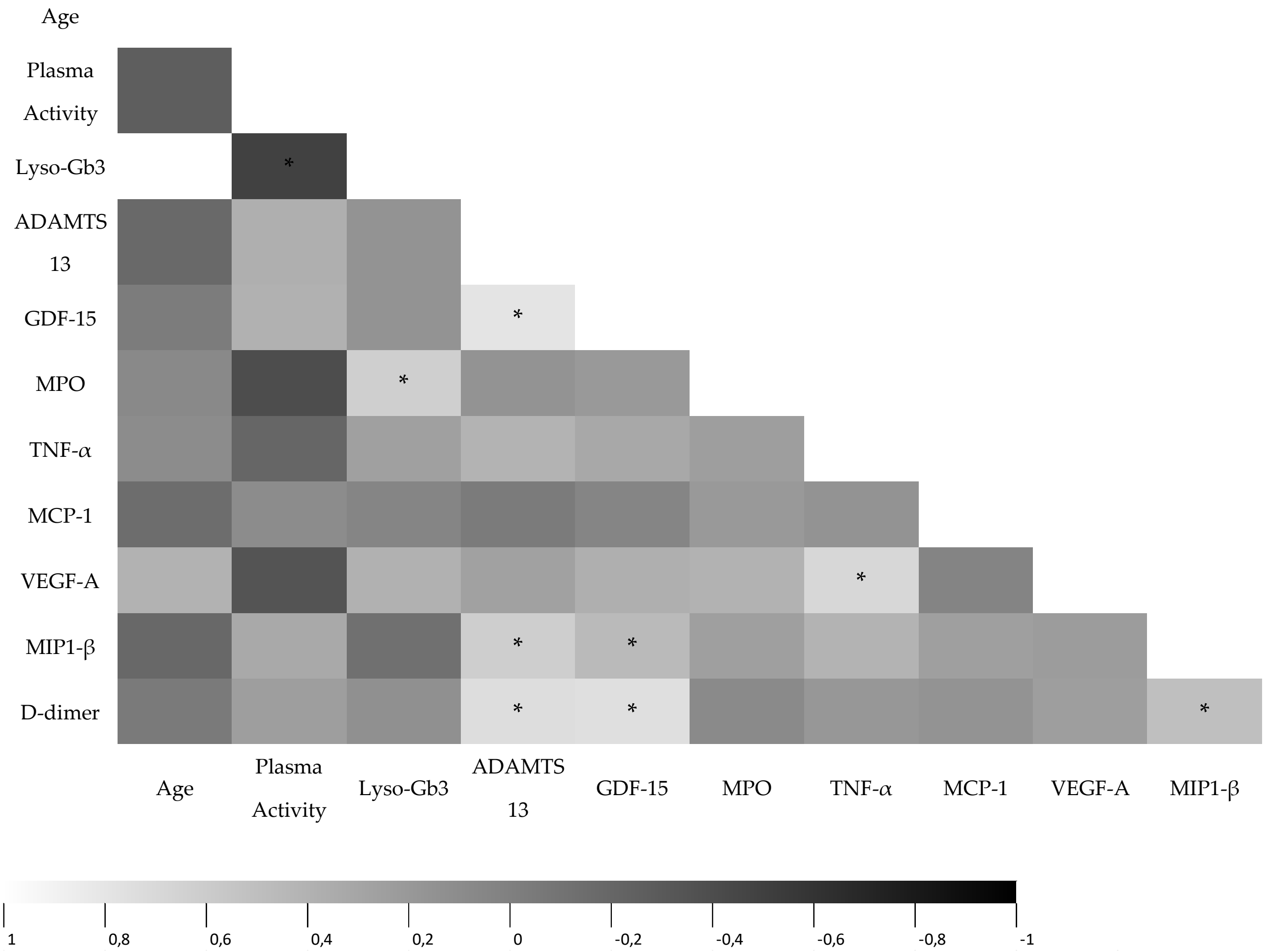

C) Female patients

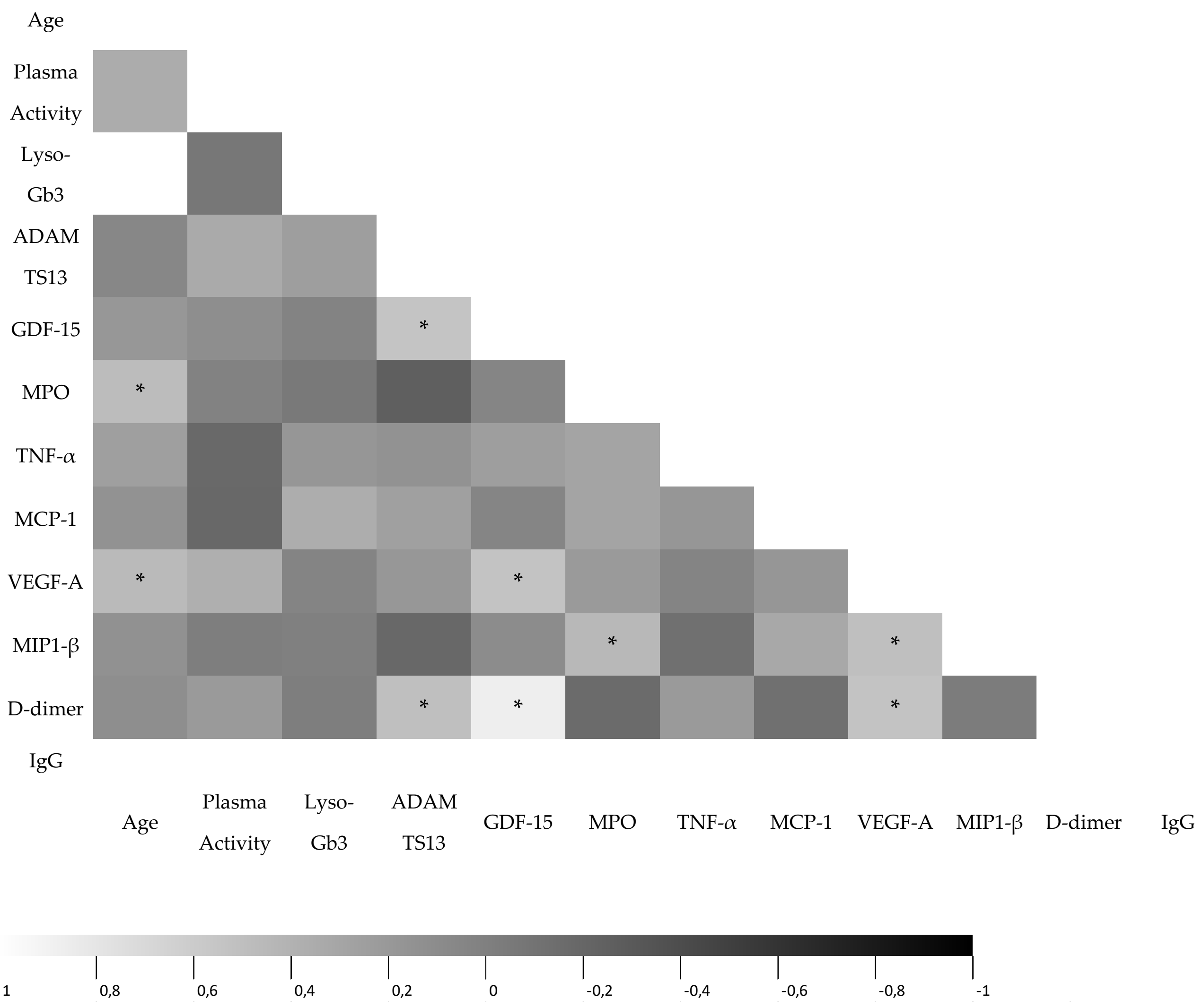

Supplementary Figure S11. Matrix of correlation of variables analyzed in plasma at basal levels independently of the treatment. *Matrix of correlation between parameters of age, immune response (IgG), fibrinolysis (D-dimer), the known biomarker (LysoGL3) and identified novel markers (ADAMTS-13, GDF-15, MPO, TNF-α, MCP-1, VEGFA, MIP-1β) biomarkers. The matrix analyzes the correlation between variables in all groups at basal time. Spearman's correlation coefficients were calculated using SPSS and significant level established for *p < 0.05. Representation model according to Gregorio et al. 2022 [1]. A) All patients, B) Males, C) Females*

SI Fig. S12

A) Basal:

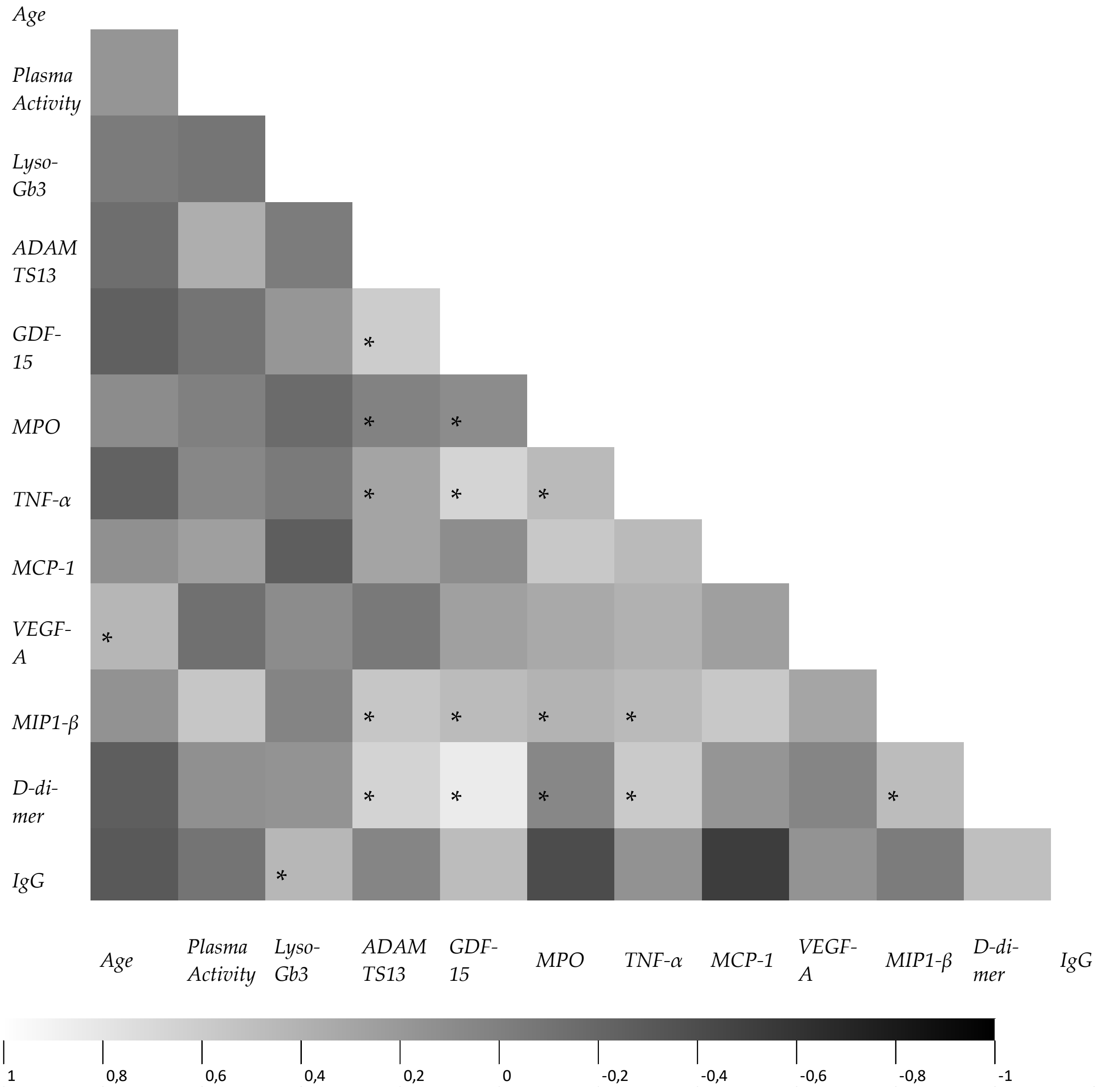

B) 6 months follow up:

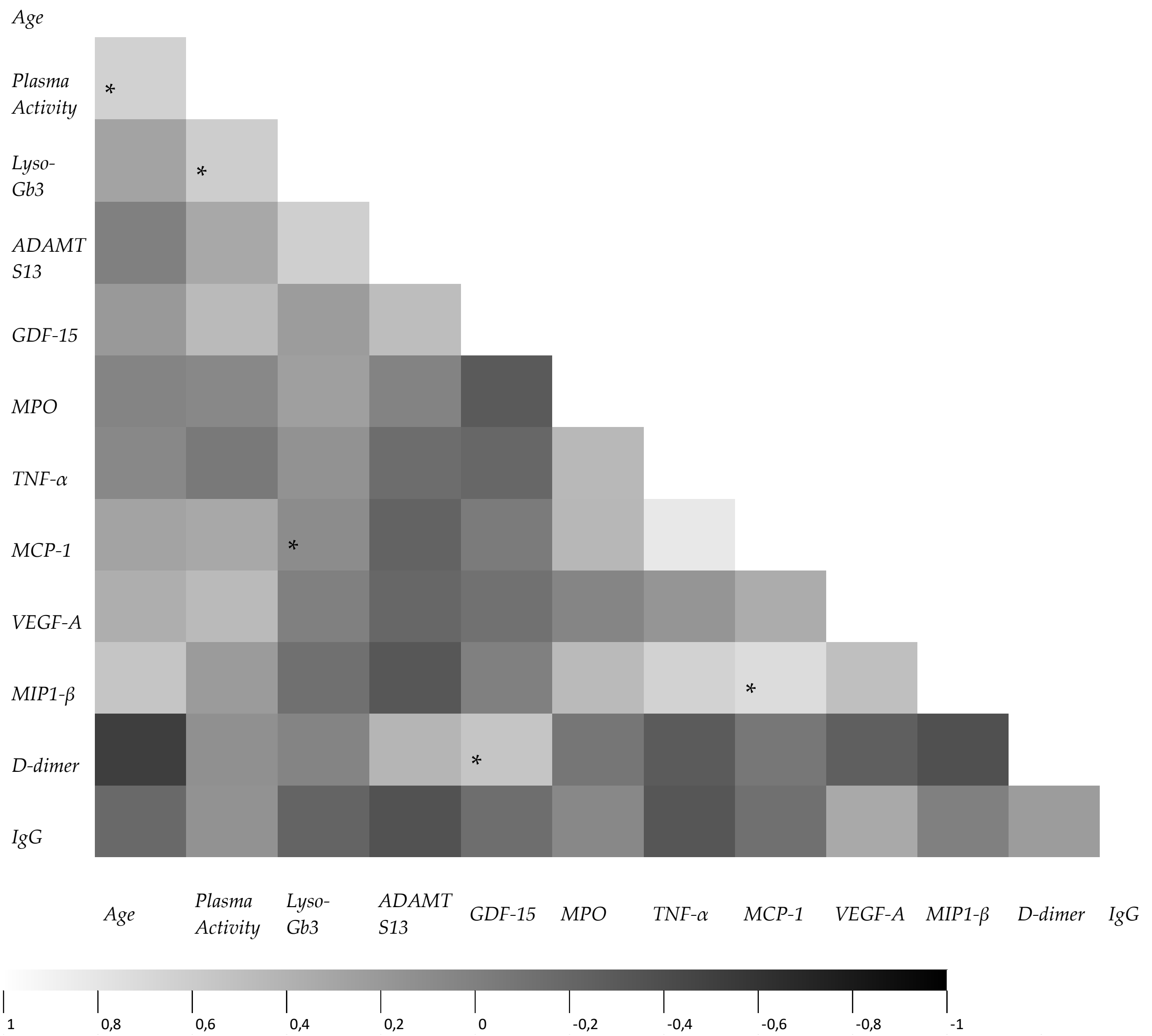
Age
Plasma Activity
Lyso-Gb3
ADAMTS13
GDF-15
MPO
TNF-α
MCP-1
VEGF-A
MIP1-β
D-dimer
IgG
*
1
0,8
0,6
0,4
0,2
0
-0,2
-0,4
-0,6
-0,8
-1

C) *12 months follow up*

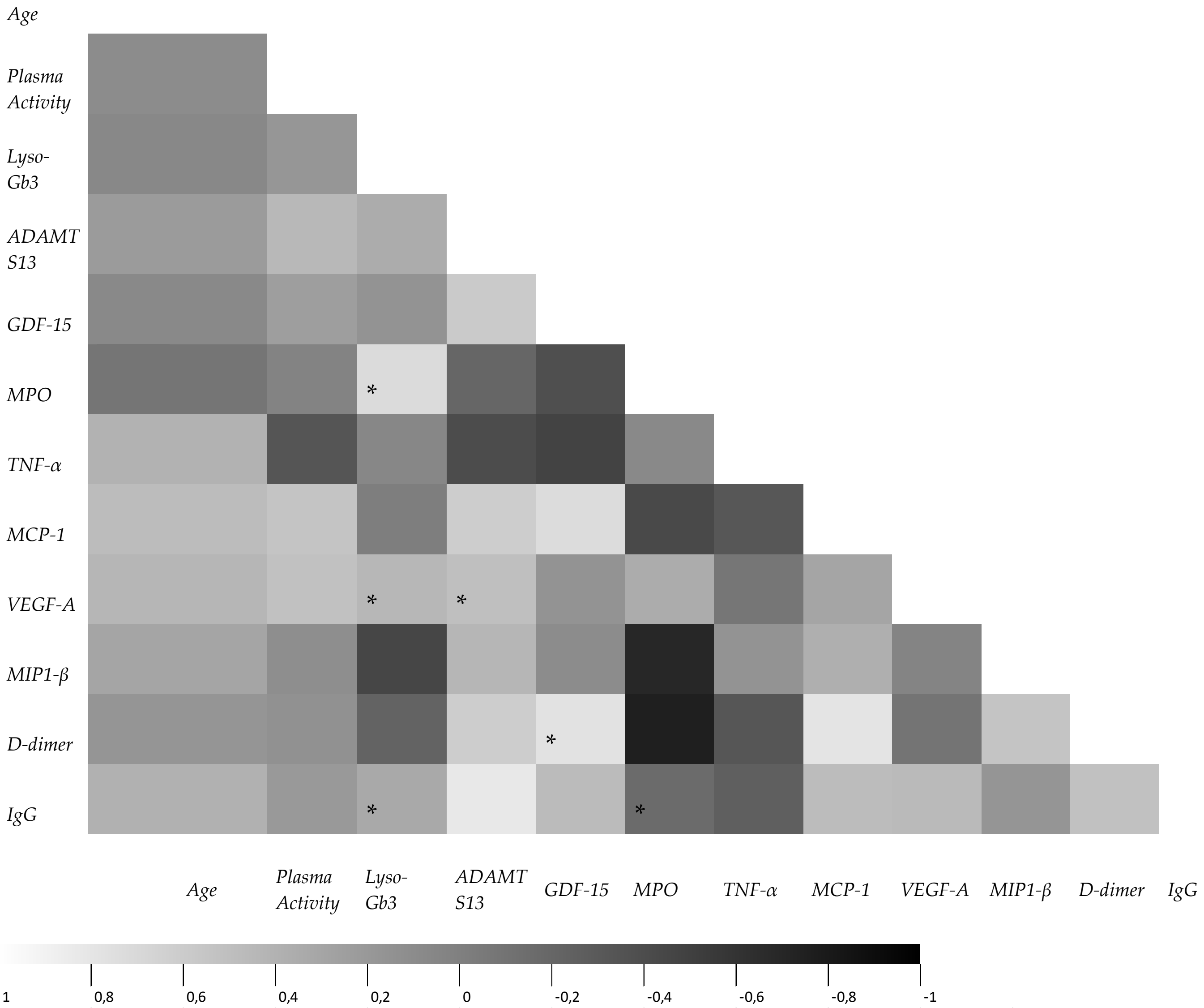

Supplementary Figure S12. Matrix of correlation of plasmatic biomarkers in ERT cohorts at all times. *Parameters of immune response (IgG), fibrinolysis (D-dimer), age, LysoGL3 and novel markers (ADAMTS, GDF, MPO, TNF, MCP-1, VEGF, MIP1B) were assessed. Spearman's correlation coefficients were used to assess the relationship between the plasma levels of the different biomarkers in ERT patients at basal levels (a), 6 months follow up (b) and 12 months follow up (c). All analyses were performed with IBM SPSS (Statistical Package for the Social Sciences) v.19. *p < 0.05. Representation model according to Gregorio et al. 2022 [1]. A) basal; B)6 months follow up; C) 12 months follow up.*

References

1. Gregório, P.C.; Biagini, G.; Cunha, R.S.D.; Budag, J.; Martins, A.M.; Rivas, L.V.; Schiefer, E.M.; Sánchez-Niño, M.D.; Ortiz, A.; Stinghen, A.E.M.; et al. Growth Differentiation Factor-15 and Syndecan-1 Are Potential Biomarkers of Cardiac and Renal Involvement in Classical Fabry Disease under Enzyme Replacement Therapy. *Kidney and Blood Pressure Research* **2022**, *47*, doi:10.1159/000521329.
